# Gel Point as Measurement of Dispersion Degree of Nano-Cellulose Suspensions and Its Application in Papermaking

**DOI:** 10.3390/nano12050790

**Published:** 2022-02-26

**Authors:** Jose Luis Sanchez-Salvador, Ana Balea, Carlos Negro, Maria Concepcion Monte, Angeles Blanco

**Affiliations:** Chemical Engineering and Materials Department, Universidad Complutense de Madrid, Avda. Complutense s/n, 28040 Madrid, Spain; josanc03@ucm.es (J.L.S.-S.); anabalea@ucm.es (A.B.); cnegro@ucm.es (C.N.); cmonte@ucm.es (M.C.M.)

**Keywords:** nanocellulose, dispersion degree, gel point, cellulose nanofibers, aspect ratio, papermaking, mechanical properties

## Abstract

The dispersion degree of cellulose micro and nanofibrils (CMFs/CNFs) in water suspensions is key to understand and optimize their effectiveness in several applications. In this study, we proposed a method, based on gel point (Ø_g_), to calculate both aspect ratio and dispersion degree. This methodology was validated through the morphological characterization of CMFs/CNFs by Transmission Electronic Microscopy. The influence of dispersion degree on the reinforcement of recycled cardboard has also been evaluated by stirring CMF/CNF suspensions at different speeds. Results show that as stirring speed increases, Ø_g_ decreased to a minimum value, in which the aspect ratio is maximum. Then, Ø_g_ increased again. Suspensions with lower Ø_g_, in the intermediate region of agitation present very good dispersion behavior with an open and spongy network structure, in which nanofibril clusters are totally dispersed. Higher stirring speeds shorten the nanofibrils and the networks collapse. Results show that the dispersion of the nanocellulose at the minimum Ø_g_ before their addition to the pulp, produces higher mechanical properties, even higher than when CNFs and pulp are agitated together. This method allows for the determination of the CMF/CNF dispersion, to maximize their behavior as strength agents. This knowledge would be crucial to understand why some industrial trials did not give satisfactory results.

## 1. Introduction

Several effective methods have been applied to disintegrate cellulose fibers into substructures with micro-or nano-size dimensions as cellulose micro/nanofibrils (CMFs/CNFs) or micro/nanocrystals [1,2,3,4]. Among them, the use of CMFs/CNFs has gained attention due to their high surface area, high strength, or their excellent barrier properties [5,6,7,8]. CMF/CNF applications have been thoroughly studied in several fields such as papermaking [9,10,11], food packaging [12,13], anti-microbial films [14], biomedical applications [15,16,17], wastewater treatments [18,19], or cement-based materials [20]. However, although their effect has been highly successful at lab scale, these data are not always replicable. This is the case of the reinforcement of recycled cardboard for packaging, where improvements above 60% in the Tensile Index has been achieved at lab scale using 3% of CNF prepared from the same raw material, pretreated with 10 mmol/g of NaClO in TEMPO-mediated oxidation and then homogenized [10,11]. However, the uncertainty of pilot trials has limited the industrial application, especially when nanocellulose was not produced on-site [21,22]. This fact is still not fully understood.

CMF/CNF gels production has been widely studied during the last decade; nevertheless, their characterization is an area still under development. Many parameters have been developed to characterize CMFs/CNFs in terms of morphology, rheological properties, composition, carboxyl or aldehyde content, nanofibrillation degree, etc. [2,5,23,24]. CMFs/CNFs have been tested in strength and coating applications, failing without any apparent reason. This suggests that some key parameters are not taken into consideration as, for example, the dispersion degree of nanofibrils before its application or the mixing of CNFs within the studied matrix [25]. Some researchers have studied the importance of dispersion and uniformity of nanocellulose on the reinforcement performance, e.g., in paper [26] or in polymeric matrices [27]. Although several methods for measuring distribution size or homogeneity, such as dynamic light scattering, turbidity, self-assembly, and shear birefringence have been reported [23], there are currently no methodologies for determining dispersion degree on the CMF/CNF suspensions despite being an important parameter for the use of nanocelluloses at industrial scale [21].

Our hypothesis is that the stirring methods used to prepare the CMF/CNF suspensions may have an impact on the entanglement network affecting the aspect ratio (AR) of CMFs/CNFs as well as the separation between the nanofibers and, therefore, their final behavior. AR determination is based on several sedimentation methodologies: on the one hand, the use of the gel point or connectivity threshold (Ø_g_) [28] and, on the other hand, the use of the differences in light transmission with time due to sedimentation [29]. In this study, the Ø_g_ method is proposed to study the dispersion of CMF/CNF suspensions and validated by the morphological characterization of the CMFs/CNFs using Transmission Electronic Microscopy (TEM) images to determine both the CMF/CNF structure and the mean diameter of the nanofibrils.

**Figure 1:**
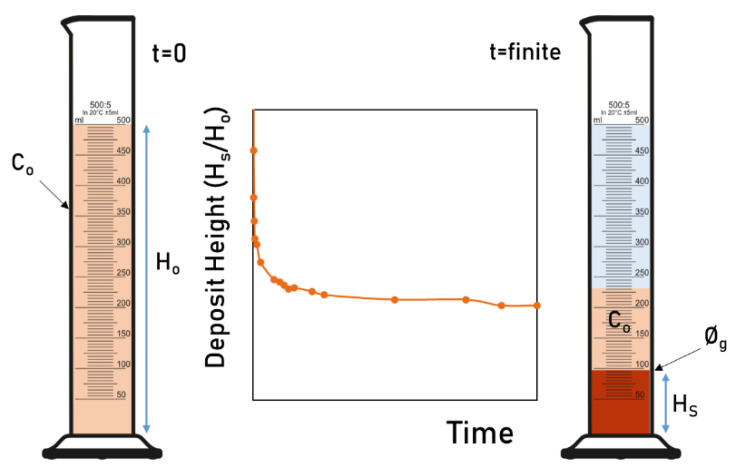
Progression of a self-supporting region with time.

Ø_g_ describes the compressibility and structure of sediments and is defined as the volume concentration of a suspension in the boundary between semi-dilute and dilute region which depends on time [6,28]. This volume fraction is also considered the lowest volume fraction, at which all primary fibres and fibre flocs are interconnected throughout the container, forming a self-supporting three-dimensional network. Below the Ø_g_ concentration, the material suspended does not contribute to the mechanical strength of the suspension [30]. Figure 1 shows the progress with time in a sedimentation experiment. During the experiment, the volume fraction on the top of the sediment is considered to be unchanged from C_o_ and, above this fraction, a clear liquid region appears [31]. As time passes, a concentrated deposit is formed in the lower part of the measured cylinder, whose volume becomes constant with time. 

Where C_o_ is the initial concentration of solids, and H_s_/H_o_ is the relation between the sediment height and the initial suspension height.

In order to adapt the Ø_g_ methodology to CNFs, it was necessary to use a dye to visualize the sedimentation line of nanofibers. Cristal violet was selected since it did not influence the sedimentation of the samples. Results showed that highly fibrillated CNFs presented sedimentation curves different from the conventional ones, requiring longer sedimentation times to obtain stable deposits than other cellulosic materials, as previously published [32].

To determine Ø_g_ values, at least five sedimentation experiments with different C_o_ are required. The curve that relates the C_o_ against the H_s_/H_o_ is plotted. Then, the derivative of the curve close to zero allows the determination of Ø_g_ as Equation (1) shows, in which Ø_g_ and C_o_ have the same units [31,33,34,35].). Two mathematical methods have been traditionally used to calculate Equation (1). The first one is the fitting to a quadratic equation without an independent term in which Ø_g_ is the first order coefficient [32,36,37], and the other is using the fitting tool CSAPS in MATLAB [28,38]. Both are tedious and time-consuming. Recently, a simplification of the Ø_g_ methodology (Equation (2)) allows to reduce the experiments labor by at least 60%, using only one measured cylinder or two if the C_o_ selected is not suitable, showing an error lower than 7% in Ø_g_ values and 3% in the calculation of the estimated AR [39].

(1) $$\emptyset_{g}=\underset{\frac{H_{s}}{H_{o}}\to 0}{\lim}\left(\frac{{\mathrm{dC}}_{o}}{d\left(\frac{\mathrm{Hs}}{\mathrm{Ho}}\right)}\right)$$

(2) ∅g(est)=Co(i)−Co(0)(HsHo(i))−(HsHo(0))=Co(i)−0(HsHo(i))−0=Co(i)(HsHo(i))

To estimate AR from Ø_g_, the Effective Medium Theory (EMT) and the Crowding Number (CN) theory [36,37] are two possible alternatives. EMT was firstly developed by Celzard et al. [40] to describe the conductivity of a material in which the particles are dispersed, for example, for spheroid graphene particles dispersed in air. AR and Ø_g_ are related by the Equations (3) and (4), according to Celzard et al. and Varanasi et al. [37,40].

(3) $$\emptyset_{g}\left(\frac{\mathrm{vol}.\%}{\mathrm{vol}.\%}\right)=\frac{9L_{c}\left(1-L_{C}\right)}{2+L_{c}\left(15-9L_{c}\right)}$$

(4) $$L_{c}=\frac{{\mathrm{AR}}^{-2}}{2{\sqrt{1-{\mathrm{AR}}^{-2}}}^{3}}\left[\ln\left(\frac{1+\sqrt{1-{\mathrm{AR}}^{-2}}}{1-\sqrt{1-{\mathrm{AR}}^{-2}}}\right)-2\sqrt{1-{\mathrm{AR}}^{-2}}\right]$$

where L_c_ is the depolarization factor of the particles.

Kerekes and Schell [41] developed the CN theory that relates the Ø_g_ and the AR of the cellulose fibers. Then, Ø_g_ was estimated by Martinez et al. [35] with a CN value of 16 ± 4, based on the analysis of positron emission tomography (PET) measurements of dilute fiber sedimentation experiments. This fact establishes a relationship between Ø_g_ and AR in Equation (5):

(5) $$\emptyset_{g}\left(\frac{\mathrm{vol}.\%}{\mathrm{vol}.\%}\right)=24/{\mathrm{AR}}^{2}$$

However, the volume fraction is more difficult to measure than the solid fraction, Ø_g_ (kg fiber/ kg suspension) as Varanasi et al. [37] have demonstrated. Therefore, the relation between Ø_g_ (vol./vol.) and Ø_g_ (wt.%/wt.%) may be expressed by Equation (6).

(6) $$\emptyset_{g}\left(\frac{\mathrm{vol}.\%}{\mathrm{vol}.\%}\right)=\frac{{Ø}_{g}\left(\frac{\mathrm{wt}.\%}{\mathrm{wt}.\%}\right)\cdot \rho_{L}\left(\frac{\mathrm{wt}.\%}{\mathrm{vol}.\%}\right)}{\rho_{F}\left(\frac{\mathrm{wt}.\%}{\mathrm{vol}.\%}\right)+{Ø}_{g}\left(\frac{\mathrm{wt}.\%}{\mathrm{wt}.\%}\right)\cdot \rho_{L}\left(\frac{\mathrm{wt}.\%}{\mathrm{vol}.\%}\right)-{Ø}_{g}\left(\frac{\mathrm{wt}.\%}{\mathrm{wt}.\%}\right)\cdot \rho_{F}\left(\frac{\mathrm{wt}.\%}{\mathrm{vol}.\%}\right)}$$

where ρ_F_ is the CMF/CNF density [37] assumed as 1500 kg/m^3^, and ρ_L_ the density of the suspension. If the CMF/CNF dose is very low, under 1 wt.%, the density of the suspension could be approximated to the water density.

In this paper, different CMF/CNF hydrogels with various fibrillation degrees have been characterized and dispersed at different stirring speed levels. The behavior of the obtained samples was analysed based on the Ø_g_ data. TEM images and AR theories have been used to validate the effectiveness of the Ø_g_ parameter to characterize the dispersions.

Based on this method, the optimal stirring-speed conditions of CNFs have been applied for an industrial application. Therefore, the effect of the dispersion degree of CNF hydrogels, before the mixture of nanocellulose with the pulp on the reinforcement of recycled old corrugated cardboard (OCC) sheets has been evaluated by measuring mechanical (bursting index, short-span compressive test index, tensile index, tear index) and physical properties (bulk and porosity) of the handsheets. In addition, the effect of disintegrating CNF and OCC together in a pulper, without a previous dispersion of CNF, was also studied.

## 2. Materials and Methods

### 2.1. Materials

CMFs/CNFs form a three-dimensional network structure within the hydrogel that is modified when the suspensions are prepared at different dispersion degrees. Four types of CMFs or CNFs have been used in this study from three different sources. The analysis of samples with different fibrillation degrees is crucial to validate the hypothesis of the study. The first sample was a microcellulose obtained from grinded cotton linters (C-CMF) with high purity cellulose (Sigma-Aldrich, St Louis, MO, USA). C-CMF were prepared without any pretreatment and mechanically fibrillated in a laboratory homogenizer PANDA PLUS 2000 manufactured by GEA Niro Soavy (Parma, Italy) using six progressive passes of homogenization from 300 to 900 bars.

Commercial recycled newspaper with an ash content of 14% was used to prepare two types of products with different fibrillation, CMF (R-CMF) and CNF (R-CNF). Both were disintegrated at 30,000 revolutions and 3.0 wt.% consistency in a pulp disintegrator (PTI, Vorchdorf, Austria). To obtain R-CMF, the cellulose pulp was refined at 5000 revolutions in a PFI mill (Hamjern Maskin AS, Hamar, Norway) and six steps of homogenization at 600 bars were applied. R-CNF was obtained by a chemical pre-treatment with 2,2,6,6-tetramethylpiperidin-1-oxyl-radical (TEMPO) according to Saito et al. [42] using 1 mmol NaBr and 10 mmol NaClO/g of pulp. Once the pulp was oxidized, a filtration cleaning process was performed using distilled water and four steps of homogenization at 600 bars have been applied.

The third raw material was obtained from *Eucalyptus globulus* ECF bleached kraft pulp, kindly supplied by Torraspapel, S.A. (Zaragoza, Spain), with 74% of cellulose and 18% of hemicellulose as its main components. The CNF (E-CNF) were obtained by TEMPO-mediated oxidation with 2.5 mmol of NaClO/g of dry pulp. Then, six steps of homogenization at 600 bars were applied. All hydrogels were stored at 4 °C at ~1 wt.% to avoid their aggregation, after adding some drops of glutaraldehyde as bactericide (5 drops/L) to avoid bacterial growth, until they were used.

The raw materials and the pretreatments used to obtain the CMFs/CNFs were selected to obtain a large variation of the properties. Dry CMFs/CNFs composition was characterized before the HPH treatment. Cellulose, hemicellulose, lignin (soluble and insoluble), extractives, and ashes were measured. Firstly, extractives of the samples were determined via Soxhlet extraction and ash content was determined via calcination according to TAPPI T204 and T211, respectively. Cellulose, hemicellulose and lignin content were obtained following NREL/TP-510-42618. 300 mg of sample was hydrolyzed for 1 h with 3 mL of 72 wt.% H_2_SO_4_. Then, 84 g of water was added in the sample and then introduced in the autoclave for one hour at 121 °C. The hydrolyzed samples were vacuum filtered, the insoluble lignin remained in the filter and the soluble part was obtained in the UV-visible spectrophotometer by measuring the absorbance of the filtrate. Hemicellulose and cellulose content were analyzed using high performance liquid chromatography (HPLC) from the filtrate after neutralization with CaCO_3_ and filtered. This process was not carried out in C-CMF due to the sample was not pretreated and directly was homogenized. CNFs/CMFs from recycled paper present a higher amount of ashes from the fillers added in the previous paper manufacture. On the other hand, the amount of cellulose is slightly reduced in the chemical pretreatment of R-CNF at the same time the insoluble lignin is also reduced. This is produced by the removal of lignin and amorphous cellulose during TEMPO-mediated oxidation. In the same way, the amount of cellulose also is slightly reduced in E-CNF respect to the initial cellulose value. However, this effect occurs to a lesser extent due to the lower amount of NaClO used in E-CNF pretreatment.

CMFs/CNFs were characterized according to Balea et al. 2019 [10] and listed in Table 1. As for the chemical parameters, the number of carboxyl groups differs greatly between the CNFs and the CMFs, the latter with almost zero content since these samples have not been oxidized in the pretreatment. The superficial cationic demand obtained show also the same trend as carboxyl groups. The TEMPO-mediated oxidation influences on the fibrillation of the samples, with a higher transmittance of these samples that reach the range of nanofibrils. On the other hand, C-CMF from cellulose powder shows a low polymerization degree in a similar way than CNFs, however the diameter size is much larger than the other samples, with a low aspect ratio value. This effect is due to the ground of the sample that breaks the cellulose chains and decreases the aspect ratio without reaching the nanofibrillation of the sample. Diameter average of the other samples is in the nanoscale, although the R-CMF show a higher number of fibers in the microscale as nanofibrillation yield indicates.

### 2.2. Methods

#### 2.2.1. Determination of Gel Point in Suspensions

**Figure 2:**
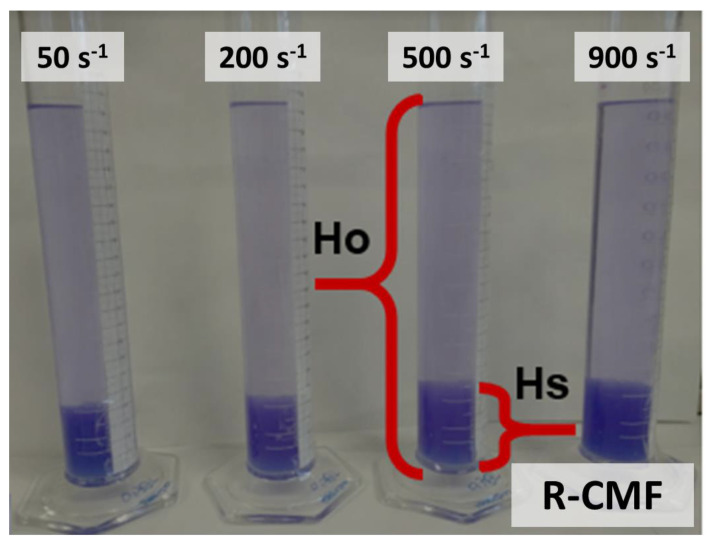
Graduated cylinders at different stirring speeds.

The dispersion of nanofibrils was evaluated before its application, according to the methodology described by Martinez et al., based on the settling of samples to calculate Ø_g_ [35]. They analyzed the relation between the sediment concentration and the compressibility effects, redefining the Ø_g_ and developing Equation (1), which later was simplified in Equation (2). To prepare sedimentation experiments, suspensions with different CNF hydrogels were prepared using deionized water and 200 µL of crystal violet 0.1 wt.% to dye the fibres [32]. Samples were agitated at several velocity gradients (G) up to 3000 s^−1^ using an overhead stirrer Heidolph RZR 2051 (Heidolph Instruments GmbH & Co. KG; Schwabach, Germany). Then, 250 mL of each suspension were settled into graduated cylinders until the sediment reached a steady value, which differs depending on the size of the fibrils, and to obtain the complete deposition of the suspension. Figure 2 shows the graduated cylinders of R-CMF stirred at different agitations.

The C_o_ was the same for all the samples to favor the comparison between the dispersion conditions. The C_o_ was chosen to obtain a sediment height of approximately 4–12% of the total height, due to the difficulty to measure the height accurately at lower sedimentation values. On the other hand, higher sedimentation heights would cause the increment of concentrations that appears in Equation (2), for which the derivative has been substituted, moves away from the limit Hs/Ho to close to zero [39].

#### 2.2.2. Transmission Electron Microscopy

To analyze the morphology of the fibers, a small sample of each CMF/CNF suspensions was collected after being stirred. They were analysed in the Centro Nacional de Microscopía (Madrid, Spain) by TEM with a JEM 1400 microscope (JEOL, Tokyo, Japan). To prepare TEM samples, CMF/CNF suspensions were diluted until 0.01 wt.%, and a drop was settled on a copper grid covered with a carbon coating and dried. Image J software was used to process the images.

#### 2.2.3. Preparation and Characterization of Reinforced Cardboard Sheets

E-CNF was used to validate the effect of CNF hydrogel dispersion on paper strength. Ø_g_ was used as a tool to determine the best conditions of papermaking application to enhance mechanical properties. Recycled OCC was used in the preparation of sheets due to the importance to increase the strength of cardboards for packaging [43]. OCC and CNF were disintegrated separately and then blended. OCC pulp was prepared through disintegration of 70 g of dry OCC in 2000 mL of tap water (3.5 wt.%) by using a pulp disintegrator according to ISO 5263-1 standard [44]. The OCC was left to soak 24 h before disintegration to favor fiber swelling. Separately, the E-CNF solutions (1 kg/m^3^) were prepared with CNF and tap water in the high-speed overhead stirrer for 10 min at several G values from 3 to 3000 s^−1^. To prepare the handsheets, OCC-CNF pulp suspension (1.0 wt.%) were prepared from both products stirred separately and mixture with a ratio 95.5 wt.% OCC/4.5 wt.% CNF. Then, cationic starch (0.5 g CS/100 g dry pulp) was added as retention agent and stirred for 30 min at low speed previous the sheet formation. Finally, pulp suspensions were prepared with basis weight of 80 g/m^2^ according to standard ISO 5269-2 by using a Rapid Köthen sheet former [45] (PTI, Vorchdorf, Austria). Handsheets were conditioned at 25 °C and 50% humidity for at least 24 h before physical and mechanical characterization. In addition, to compare the effect of OCC and CNF disintegrated by separate or together, OCC and CNF were stirred at the same time in the pulper at 3000 s^−1^ for 10 and 60 min (30,000 and 180,000 revolutions) with the same rate CNF/OCC as previously.

To characterize the handsheets, mechanical properties were calculated using the average grammage of handsheets. Then, bursting strength index (kPa·m^2^/g), SCT index (N·m/g), tensile strength index (kN·m/kg) and tear index (mN·m^2^/g) were evaluated. Tensile strength was measured in an MTS Criterion Mode 43 from MTS Systems Corporation (Eden Prairie, MN, USA), following ISO 1924-3 standard [46]. Bursting strength was determined using a Messmer Büchel digital hydraulic board burst tester (Veenendaal, Netherlands) according to ISO 2759 standard [47]. A short span compression tester (Messmer Büchel, Veenendaal, Netherlands) was used to measure the short-span compressive test (SCT) according to TAPPI T826 standard [48]. Finally, tearing resistance was measured with a Tearing Tester from Lorentzen & Wettre (Stockholm, Sweden) according to ISO 1974 standard [49]. Physical properties measured were porosity, basis weight, and thickness. Porosity (μm/Pa·s) was evaluated in a Bendtsen Porosity Tester number 8699 from Andersson & Sørensen (Copenhague, Denmark) according to ISO 5636-3 [50]. Basis weight and thickness were determined according to ISO 536 and ISO 534, respectively [51,52]. Finally, bulk (cm^3^/g) indicates the thickness in relation to the basis weight.

## 3. Results and Discussion

### 3.1. Evaluation of the Dispersion Degree of CMF/CNF Suspensions

Ø_g_ results were obtained by sedimentation of the CMFs/CNFs until the formation of a stable deposit. In the case of R-CNF and E-CNF with smaller sized fibers, the clear zone at the top of the cylinder is not formed at the low concentrations required for Ø_g_. This fact is due to the small size of fibers and a slow sedimentation, not allowing the distinction of the clear and coalescence layer at short times [32]. Therefore, the sedimentation curve obtained is quite different to the conventional one and only the interphase that indicates the formation of deposits is observed, in the base of the cylinders. At the beginning, the deposits increase over the time due to the compaction of part of material from the coalescence layer until all material has a compaction grade enough to be visible. In this point, we observe a clear separation of the compacted material that continues to compress until the sample has completely sedimented [32]. The height of this layer decreases until the height does not vary with time, and then, Ø_g_ is calculated. On the other hand, C-CMF and R-CMF show a conventional sedimentation.

**Figure 3:**
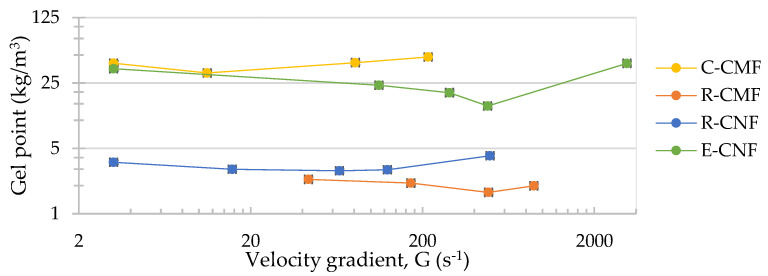
Gel point vs. stirring speed.

**Figure 4:**
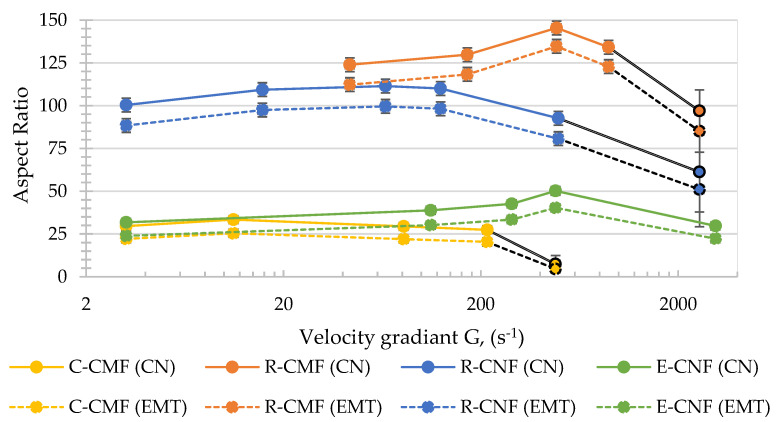
Aspect ratio of the four hydrogels at different stirring speed according to Crowding Number (CN) and Effective Medium Theory (EMT) theories.

Figure 3 shows the Ø_g_ of the different CMF/CNF hydrogels at different stirring speeds. To facilitate the study of dispersion, Equation (3) was used to simplify the experimental labour using a unique C_o_, the same for all stirring speeds but different between hydrogels, having as long as possible a H_s_/H_o_ from 4% to 12%. C_o_ used were 1.5 kg/m^3^ for C-CMF, 0.15 kg/m^3^ for R-CMF, 0.25 kg/m^3^ for R-CNF and 1 kg/m^3^ for E-CNF. The use of the same C_o_ to calculate the Ø_g_ does not allow its estimation with precision at the higher speed of C-CMF, R-CMF, and R-CNF, since the sediment height was very scarce. Therefore, these values were not shown in Figure 3 although they would be used in the estimation of AR in Figure 4. Increasing the velocity gradient, we can observe a decrease in the Ø_g_ decrease until a minimum value, then at a certain velocity gradient, Ø_g_ increase again. In those samples that presented a very low deposit at high speed (2500 s^−1^ for R-CMF and R-CNF and 500 s^−1^ for C-CMF) the Ø_g_ should be higher than the last value calculated in Figure 3 for each hydrogel.

To explain the possible modifications on the CMF/CNF suspensions with the agitation, the AR is calculated by the Equations (3)–(6) to obtain the relationship between both parameters, using the two theories, CN and EMT, and setting the AR as the independent variable according to Equations (7) and (8). Figure 4 shows AR values of the four hydrogels at the different stirring speed conditions using both theories. In this figure, the AR of C-CMF, R-CMF, and R-CNF stirred at high stirring speed was possible to be graphed although with a large accuracy error, so its representation is drawn in black.

(7) $$\mathrm{AR}\left(\mathrm{EMT}\right)=3.61\cdot {\left(\frac{\emptyset_{g}\left(\frac{\mathrm{kg}}{m^{3}}\right)}{1000}\right)}^{-0.567}$$

(8) $$\mathrm{AR}\left(\mathrm{CN}\right)=5.98\cdot {\left(\frac{\emptyset_{g}\left(\frac{\mathrm{kg}}{m^{3}}\right)}{1000}\right)}^{-0.5}$$

In all hydrogels studied, AR has a similar trend with a maximum at intermediate stirring speeds. At low agitations, water and hydrogel are hardly mixed, but the crossed networks of fibrils start to separate due to the shear forces at higher stirring speeds. In addition, in E-CNF hydrogel in which gelation has occurred after homogenization, clusters begin to break. Then, it is observed that increasing stirring speed, an increase in H_s_/H_o_ is produced that indicates the E-CNF structure is more open and spongier with the dissolution of clusters. The fact that relative height was in the denominator of Equation (2) makes that Ø_g_ decrease during this first period until a certain velocity gradient value, in which the H_s_/H_o_ is maximum. Then, at higher speeds, H_s_/H_o_ decreased because the hydrogen bonds between fibrils would break them and, therefore, the networks would be destroyed. In addition, the mechanical forces during agitation would shorten the length of the fibrils that are individual separated, compacting them in the base of the graduated cylinders and, as a consequence, increasing Ø_g_.

However, the optimal stirring speed to obtain as many dispersed fibers as possible without breaking the network is different for each hydrogel. Analyzing them one by one, C-CMF show the highest Ø_g_ studied (Figure 3), due to cotton that was ground in powder obtaining the lowest aspect ratio during all agitation stages, as Figure 4 shows. The high crystallinity of the sample with a high rigidity makes the sample not produce a network of fibers without an increase in the sponginess with the stirring. The AR in C-CMF is almost invariable with a maximum obtained at very low agitation conditions, with a velocity gradient around 10 s^−1^ associated to the initial agglomeration of the sample. This hydrogel without any pretreatment contains mainly microfibrils with scarce branches from the primary structure due to the powder state of the sample, so the crossover network is hardly possible and at low agitation the effect of fiber breakages is already evident. However, moderate agitation produces a strong shortening of the fibers as aspect ratio shows. On the contrary, the R-CMF shows a higher number of branches around the cellulose backbone due to the pulp was refined as pretreatment before the homogenization, this mechanical treatment produce an increase in the AR compared with other pretreatments as TEMPO-mediated oxidation or enzymatic hydrolysis [53]. A greater crosslinking of networks as in R-CMF would produce, at low agitation speeds, the increase in the sponginess stretching the networks as it shakes. A higher velocity gradient is required to obtain the spongier network, above 500 s^−1^. Then, the high hydrodynamic conditions would produce the breakage of refined microfibrils separating them from the main structure and at the same time the shortening of fibrils.

As for CNF hydrogels, R-CNF obtained from the same raw material than R-CMF but pretreated with TEMPO-mediated oxidation instead of refining, shows a minimum Ø_g_ around 50–100 s^−1^, less agitation than R-CMF. This is because TEMPO-mediated oxidation produces electrostatic repulsion between microfibrils and the breakages of cellulose chains, favoring the fibers separation with the breakage of primary structures. Both effects would reduce the crossover between networks and, therefore, at lower stirring speeds the breakage of the fibers is observable. Finally, the other CNF hydrogel, E-CNF with an almost total nanofibrillation, shows the minimum Ø_g_ around 500 s^−1^. Despite the higher nanofibrillation yield of E-CNF, the number of carboxyl groups is less than R-CNF due to a lower NaClO content in the TEMPO-mediated oxidation, decreasing the repulsion of fibrils. This fact would produce less breakage and separation of fibers and, therefore, E-CNF keep a greater crosslinking of the fibers that are more difficult to separate mechanically requiring more energy. In addition, in E-CNF is not produced a great variation of deposits at very high stirring speeds, probably due to the almost total nanofibrillation of the fibers that prevents a great variation of heights from occurring.

### 3.2. Validation of Gel Point Methodology to Quantify Nanocellulose Dispersion

**Figure 5:**
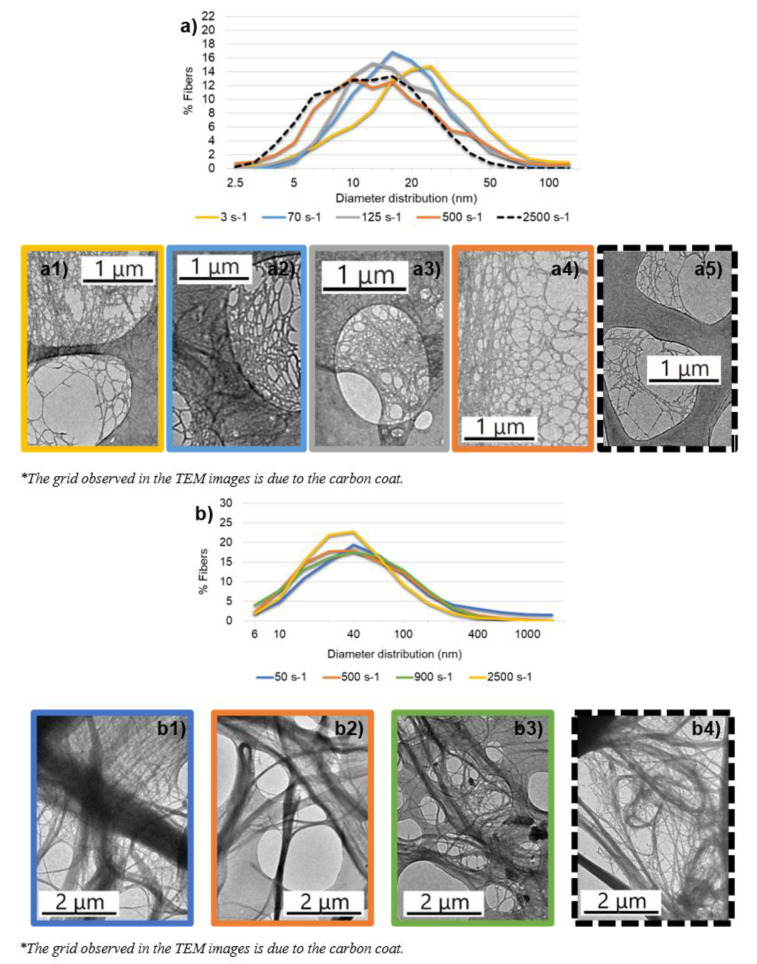
CMF/CNF diameter distribution and TEM images at different stirring speeds: (**a**) R-CNF diameter distribution; (**a1**) 3 s^−1^; (**a2**) 70 s^−1^; (**a3**) 125 s^−1^; (**a4**) 500 s^−1^; (**a5**) 2500 s^−1^; (**b**) R-CMF diameter distribution; (**b1**) 50 s^−1^; (**b2**) 500 s^−1^; (**b3**) 900 s^−1^; (**b4**) 2500 s^−1^.

To verify the shortening of the fibrils and the AR, the two hydrogels from the same raw material were selected in order to see the effect of refining and TEMPO pretreatments on the dispersion degree. TEM images of R-CNF and R-CMF were taken at different stirring speeds and magnifications from 400× to 10,000×. Diameter distributions in logarithmic scale are presented in Figure 5 together with a TEM micrograph of each hydrogel at different agitation conditions.

**Figure 6:**
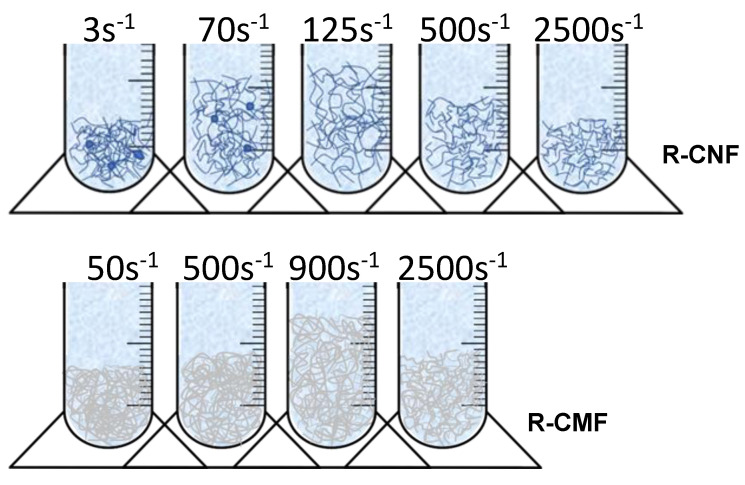
Representations of fibril network in the deposit of the graduated cylinders at increasing agitation speeds.

The differences between both hydrogels are related to the pretreatments used to obtain the hydrogels, which produce the variation of fiber size. R-CNF has a higher number of fibers in the nanometric scale due to the more intensity of chemical pretreatment than R-CMF. When the velocity gradient is low, R-CNF and R-CMF are not well dispersed in the solvent, as part of the fibrils are positioned together, and if they do not separate with more agitation, they settle as a single particle, forming aggregates or clusters. On the other hand, when the agitation is higher than that performed in the minimum Ø_g_, the fibrils network is destroyed due to the mechanical forces and the fibrils are compacted in the base of the graduated cylinders as the representation of fibre network disposition shows in Figure 6. Moreover, Table 2 shows statistical parameters of the diameter fibers including the number of samples analyzed in TEM images, the geometric mean with a 95% confidence interval, the median of each sample (to avoid the deviation of the statically tails) and the 95th percentile to evaluate the diameter of the higher fibers discarding values that may be exceptional. Finally, the length of fibrils was calculated from the AR values and the geometric mean diameter.

Analyzing both samples separately, R-CNF, with a high homogeneity of the fibrils due to TEMPO pretreatment, show at low velocity gradient (~3 s^−1^) the initial gel state of the hydrogel. At this low agitation speed, R-CNF is not well dispersed with clusters in the suspension. The nanofibrils are not effectively separated and the surface area low with the highest diameter geometric mean. Increasing velocity gradient up to 70 and 125 s^−1^, the lowest Ø_g_ are obtained and the length of fibers barely changes but the number of nanofibers with high diameter (>30 nm) decreases, indicating that clusters disappeared and the network is slightly spongier. Since the length of the fibers is maintained and the mean diameter decreases, the highest AR is obtained. Higher gradients as 500 s^−1^ produce an increase in Ø_g_ associated with the shortening of fibers and, to a lesser extent, a reduction in diameter. Extreme agitation (around 2500 s^−1^) almost halves the length of the fibers and sedimentation deposits are very compact, with the reduction of the percentile 95th below 30 nm.

R-CMF shows the same trend as R-CNF but at different stirring speeds. Low agitation (50 s^−1^) shows the higher length as the same time the higher diameter due to clusters and fibers not separated. Then, with the intermediate agitation (500 s^−1^), the minimum Ø_g_, fiber length is almost maintained but shows a drastic decrease in the diameter distribution due to the separation of the biggest fibers producing a spongy network. The effect of refining produces the separation of the microfibrils but not their breakage, resisting a higher speed than R-CNF pretreated by TEMPO mediated oxidation in which there are less bundles of fibers around the primary structures that allow the creation of networks. However, as in R-CNF, higher velocity gradients than the minimum Ø_g_ (around 900 s^−1^) show the break of the open network with the shortening of the length of nanofibrils while the diameter is maintained. This effect is also observed with an extreme agitation that halves the fiber length. In short, the agitation (stirring speed) at which the minimum Ø_g_ is produced for each CMF/CNF hydrogel would be the best agitation conditions to avoid aggregation of CMF/CNF on the one hand, and the shortening of the fibrils on the other.

### 3.3. Effect of CNF Dispersion on the Mechanical and Physical Properties of Paper

**Figure 7:**
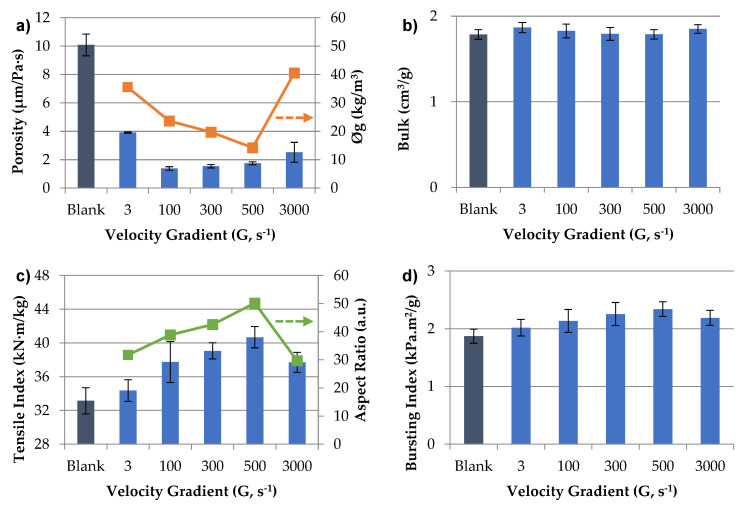

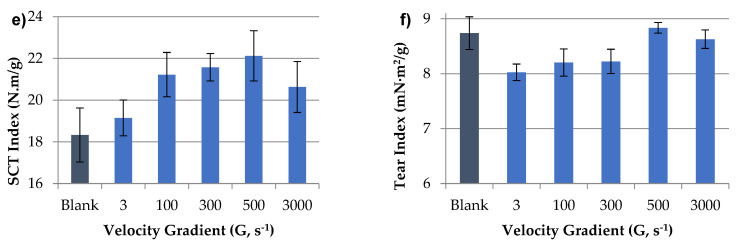
Mechanical and physical properties of handsheets prepared with OCC disintegrated for 10 min (3000 s^−1^) and CMF dispersed at different speeds for 10 min: (**a**) Porosity and Gel Point; (**b**) Bulk; (**c**) Tensile Index and Aspect Ratio; (**d**) Bursting Index; (**e**) SCT Index; (**f**) Tear Index.

**Figure 8:**
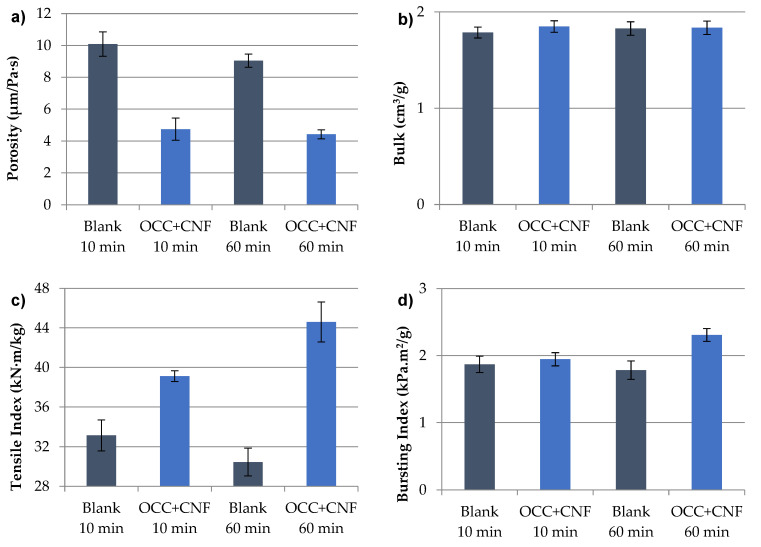

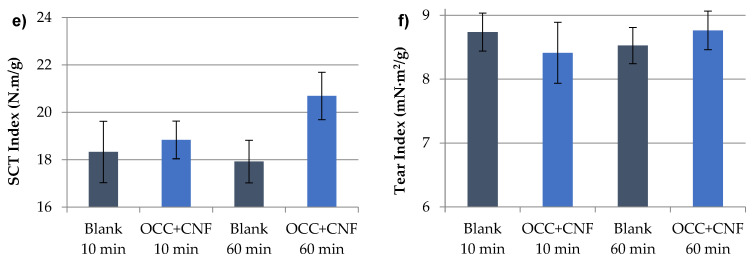
Mechanical properties of handsheets prepared with CNF and OCC stirred together at different speeds: (**a**) Porosity; (**b**) Bulk; (**c**) Tensile Strength Index; (**d**) Bursting Index; (**e**) SCT Index; (**f**) Tear Index.

The effect of CNF dispersion degree before being added to the OCC pulp disintegrated has been studied on the reinforcement of cardboard. In addition, the effect of the dispersion of CNF and the disintegration of OCC pulp at the same time has also been studied. E-CNF dispersed at different levels, the same used to determine the minimum Ø_g_ (Figure 3), has been studied before the addition in the OCC pulp that was previously disintegrated at 30,000 revolutions (around 3000 s^−1^) in a pulp disintegrator. Figure 7 shows physical (porosity and bulk) and mechanical properties (tensile, bursting, SCT and tear indexes) of the handsheets prepared when CNF was stirred separately. In addition, Ø_g_ and AR were included in Figure 7a,c, respectively, to favor the comparison of the samples. On the other hand, Figure 8 shows the results obtained when the dispersion of CNF and disintegration of OCC was carried out at the same time.

OCC blank has the highest Bendtsen porosity of 10.1 µm/Pa·s. Substituting 4.5 wt.% OCC by CNFs previously stirred and added after OCC pulping, porosity is reduced in more than 60% in all cases. This value depends on the agitation speed of CNFs, with a lower reduction at 3 s^−1^, due to CNFs not being well dispersed yet. However, at 100 s^−1^ it is produced the higher reduction of the porosity up to 1.4 µm/Pa·s that represents a reduction of more than 86%. This fact suggests a higher occupation of pores when CNFs are applied and well dispersed in the handsheets [54,55]. This porosity is almost maintained with a slightly increase until 500 s^−1^. Then, porosity increases again at 3000 s^−1^ until 2.5 µm/Pa·s. This fact is produced at the same time Ø_g_ increases again, due to the shortening of the nanofibers and the break of the network leading to a decrease in the occupation of pores. In addition, a decrease in the CNF size could produce a higher loss of CNFs during the production of the handsheets in the sheet former, moving a higher CNF propertion to the process water. As for the other physical property tested, the bulk, all samples studied have the same result as Figure 7b shows, indicating the replace of a low content of OCC by CNFs do not have influence on the thickness neither the basis weight of the handsheets.

Moving to mechanical properties, tensile strength is studied in Figure 7c. OCC blank has a tensile index of 33.1 kN·m/kg. When CNFs are stirred at several speeds, the tensile index increases progressively with the stirring speed until 40.7 kN·m/kg, when CNFs are stirred at a velocity gradient of 500 s^−1^, in which the Ø_g_ is minimal with the highest AR. It is produced an increase of 23% respect to blank and 18% compared with the sample with CNFs stirred at the low speed. This fact is due to the progressively separation of the CNF clusters from the gel with the agitation, with a more opening CNF network and better homogeneity in the suspensions and then a better distribution of the fibers in the final matrix, increasing the strength. However, an excess on the velocity gradient, above the minimum Ø_g_ (3000 s^−1^), does not improve the results with a tensile index of 37.7 kN·m/kg and an increase of 13.7% respect to blank. This fact may be due to the break of the fibers in pieces, reducing their impact to cover the pores of the fibers and the ability to interlace the fibers of the matrix reducing the tensile strength.

As other researchers have demonstrated, the bursting index (Figure 7d) shows the same trend as the tensile index [56]. OCC blank has a bursting index of 1.87 kPa·m^2^/g. Handsheets with CNFs increase progressively until a gradient velocity of 500 s^−1^, with an increase of 25% in the bursting strength (2.34 kPa·m^2^/g). However, a higher speed makes an increase in burst strength of only 17%. The same occurs with compression strength, measured with the SCT index in Figure 7e. The higher SCT is obtained when the CNFs are stirred at 500 s^−1^, with an increase of 21% respect to the blank, then the SCT Index is reduced more than the obtained with 100 s^−1^. Finally, the tear index is evaluated in Figure 7f with a blank value of 8.74 mN·m^2^/g. The addition of CNFs stirred at 3 s^−1^ decreases the tear index in 8.2%. This reduction is also observed in other studies, when CNFs are applied in bulk to prepared handsheets due to the reduction of the length of the fibers in the matrix making the tearing easier [10,57]. Low stirring speeds do not improve the results of blank until CNFs are stirred at 500 s^−1^, maintaining tear index of blank with a slightly increase of 1.1%. This effect could be due to a good disposition of the CNFs in the OCC matrix, avoiding their aggregation and the tearing weakness. Extreme agitation of CNFs, 3000 s^−1^, produces only a little decrease in the tear index but also maintained the blank value. In summary, looking at all properties together, the optimal conditions to improve mechanical properties of recycled cardboard were CNFs stirred at 500 s^−1^ rpm with the best results in all mechanical properties studied and maintaining bulk and the tear of the OCC matrix without CNFs. These conditions coincide with the minimum Ø_g_ of E-CNF (around 500 s^−1^).

Besides the importance of dispersing CNFs adequately to prepare the handsheets, OCC disintegration is also a key factor to analyze. For that, the influence of OCC disintegration and CNF dispersion in the same pulper or separately at different stirring speeds has been studied. Figure 8 shows physical and mechanical properties of handsheets prepared with OCC and CNFs disintegrated and dispersed, respectively, at the same time in the same pulper at 3000 s^−1^ for 10 min and 60 min. Blank prepared only with OCC and stirred 10 min is the same as in Figure 7, being able to compare the results of stirred OCC and CNF together or separately.

Bendtsen porosity of Figure 8a shows the variation of adding CNF in the pulp matrix in both speeds. A higher stirring speed does not produce a high diminution of porosity, being ineffective a higher time of disintegration. In addition, both samples are more porous than when OCC and CNFs are stirred separately. This fact could be due to in agitation separately the CNFs are placed more superficial whereas in the stirring together both materials mix with each other in a higher extent. In respect to bulk, as in Figure 7, there are not differences in respect to blanks without CNFs.

As for the mechanical properties, tensile, bursting, and SCT indexes show the same trend (Figure 8c–e). In the three indexes, there is a decrease in these properties when the blank is stirred for 60 min due to a higher break of the fibers in the disintegrator. As for the sheets with OCC and CNF in both times of stirring, they have an increase in the properties respect to the blanks, even more after 60 min of agitation. Among the results stand out the tensile index of OCC and CNF stirred together for 60 min with 44.6 kN·m/kg. This property has the highest difference with the tensile of OCC (10 min) and CNF (500 s^−1^) stirred separately with 40.7 kN·m/kg (Figure 7c), although if we compare with the OCC and CNF stirred together but only 10 min (39.1 kN·m/kg) we see that the OCC disintegration and CNFs dispersion separately is still better. As for the bursting and SCT indexes, the separated stirring of OCC and CNFs (500 s^−1^) shows better properties than the obtained together independently the time selected (Figure 8d,e). This fact indicates that, as in porosity, the mix of CNF with OCC after its disintegration favors the mechanical properties of the sheets, due to a more external situation of CNF whereas a more blended situation reduces the efficiency of CNF that is more mixed in the bulking agent. Finally, the tear index in Figure 8f indicates that this property barely has influence in the handsheets when they are prepared with OCC and CNFs at the same time respect to the blanks, obtaining the same maintenance of the properties as stirring OCC and CNFs separated at the optimal speed. Therefore, to sum up among the samples with OCC stirred for 10 min, the agitation separately of OCC and CNFs, these dispersed in the optimal conditions in which the minimum Ø_g_ is obtained (500 s^−1^), producing the best results in tensile, bursting, and SCT indexes, maintaining the tear index, and reducing notably the porosity. Comparing these results with those obtained after 60 min of stirring the OCC and CNFs together, the only property that increases is the tensile index, such that a longer disintegration time would be discarded due to the higher energy consumption.

## 4. Conclusions

Dispersion of CMF/CNF hydrogels has been quantified for the first time based on the Ø_g_ methodology and validated by the morphological characterization with TEM. The important effect of this key parameter has been developed to facilitate the optimal use of CMFs/CNFs in applications at industrial scale. The stirring speed required to obtain the maximum expanded CMF/CNF network without clusters and without breaking down of the network depends on the fibrillation degree and the charge of each CMF/CNF hydrogel. Increasing stirring speed produces a decrease in the Ø_g_ that indicates the CMF/CNF structure is more open and spongier. However, too high levels of agitation produce an increase in the Ø_g_ because the nanofibrils are broken and separate from the network by the high mechanical forces from the stirring of the suspensions; the CMF/CNF network collapses, which results in the compaction of the nanofibrils.

Based on the results, it is concluded that the optimum stirring speed for a given CMF/CNF sample is the one corresponding to the minimum Ø_g_, which in turn corresponds to the maximum AR. At the minimum Ø_g_, the aggregation of nanofibrils is avoided as well as the reduction of their surface area, observed when CMF/CNF hydrogels are not well dispersed, and the shortening of the fibers is minimized. This value can be easily obtained on-site and will allow for the optimization of industrial CMF/CNF applications. However, the optimal dispersion of each CMFs/CNFs must be studied separately, due to depending on several parameters such as the fibrillation, treatments in the production, or composition. This technique has been tested in a real application, as in the case of the reinforcement of the mechanical properties of cardboards. When E-CNF suspensions are dispersed at different stirring speeds and then added to the OCC matrix disintegrated separately, the best mechanical properties were obtained when CNFs were dispersed at the minimum Ø_g_. At this dispersion degree, there is not an excess break of the fibers nor the presence of clusters or agglomerations that allow the ability to interlace the fibers of the matrix with the CNFs. In addition, this configuration also presents better properties than when the OCC was disintegrated at the same time that the CNFs were dispersed. The separate agitation of OCC and CNFs allows CNFs to be placed more superficially, covering the pores in the OCC network, without an excessive mixture of both components avoiding the CNFs being located more internally, reducing the effect of covering pores and taking part of the own matrix.

## Figures and Tables

**Table 1:** Characterization of CMFs/CNFs.

	C-CMF	R-CMF	R-CNF	E-CNF
	Dry composition			
Cellulose (%)	>99.9	56 ± 1	50 ± 1	72 ± 1
Hemicellulose (%)	-	13 ± 1	18 ± 1	18 ± 1
Soluble lignin (%)	-	4.3 ± 0.5	10.0 ± 0.5	6.0 ± 0.5
Insoluble lignin (%)	-	12.5 ± 0.5	5.3 ± 0.5	-
Extractives (%)	-	1.8 ± 0.1	2.0 ± 0.2	0.3 ± 0.1
Ashes (%)	<0.1 *	12.5 ± 0.3	14.0 ± 0.5	3.0 ± 0.5
	Chemical parameters			
Carboxyl Groups (mmol/g)	0.06	0.07	0.81	0.59
Superficial cationic demand (meq/g)	0.06	0.04	0.62	0.80
	Morphological parameters			
Transmittance 400 nm (%)	2.1	1.8	15.4	83.5
Transmittance 800 nm (%)	9.2	8.7	35.7	94.8
Polymerization Degree (monomeric units)	229	703	201	440
Nanofibrillation Yield (%)	<5	39	78	89
Diameter (average)	~5 μm	44 nm	19 nm	28 nm

* In accordance with the instructions of the manufacturer.

**Table 2:** Statistical parameters of diameter distribution of CMF/CNF samples from TEM images and estimation of aspect ratio and length of fibers.

Velocity Gradient (s^−1^)	Geometric Mean of Diameter (nm)		Diameter Median (nm)	Diameter D(0.95) (nm)	Number of Samples Measured (Fibers)	Length (µm)	
	95% Confidence Interval	Mean				CN	EMT
R-CNF							
3	(17.4, 20.3)	18.8	18.9	50.7	261	1.89	1.67
70	(14.4, 16.5)	15.4	15.3	38.0	237	1.72	1.53
125	(14.0, 15.9)	14.9	14.1	39.0	311	1.64	1.46
500	(11.8, 13.6)	12.6	11.8	40.7	366	1.17	1.02
2500	(9.9, 11.5)	10.7	10.9	27.4	242	<0.9 *	<0.8 *
R-CMF							
50	(39.0, 49.5)	43.9	40.0	379	316	5.44	4.93
500	(29.5, 36.4)	32.8	31.5	145	259	4.77	4.41
900	(29.7, 35.3)	32.4	33.9	136	366	4.34	3.98
2500	(27.4, 32.4)	29.8	26.8	111	295	<3.2 *	<2.9 *

* Gel point at 2500 s^−1^ was not determined with precision due to the low deposits obtained. Therefore, a minimum value to obtain the maximum length of fibrils at that stirring speed is assumed.

## Data Availability

Not applicable.
